# Block copolymers for designing nanostructured porous coatings

**DOI:** 10.3762/bjnano.9.218

**Published:** 2018-08-29

**Authors:** Roberto Nisticò

**Affiliations:** 1 Department of Applied Science and Technology DISAT, Polytechnic of Torino, C.so Duca degli Abruzzi 24, 10129 Torino, Italy

**Keywords:** block copolymers, coatings, materials science, porous materials, self-assembly

## Abstract

Highly ordered porous coatings find applications in many fields, such as nanotechnology, microfluidics and nanofluidics, membrane separation, and sensing. In recent years, there has been great interest regarding the synthesis of isoporous and well-ordered (in)organic coatings for the production of highly selective functional membranes. Among the different strategies that have been proposed to date for preparing these porous thin coatings, one simple route involves the use of self-assembled amphiphilic block copolymers either as the porogen (acting as sacrificial templating agents for the production of inorganic architectures) or as a source of the porogen (by self-assembly for the production of polymeric substrates). Therefore, an extended discussion around the exploitation of block copolymers is proposed here in this review, using polystyrene-*block*-polyethylene oxide (PS-*b*-PEO) as the model substrate, and critical points are highlighted.

## Review

### Introduction

Porous materials have received much attention because they can be successfully applied in many fields, such as nanotechnology, membrane separation, microfluidics and nanofluidics, sensing, catalysis, and biomedicine [1–5]. The manufacture of well-ordered devices at the nanometer level requires detailed control in terms of structural organization, thus introducing the concept of “matter manipulation” at the nanometer scale [6–7]. According to the literature, several methods have been proposed for the production of highly ordered porous nanostructured materials and/or coatings, which can be classified into one of the two classical routes: the bottom-up or the top-down approach [8–10]. In particular, a “top-down” approach relies on the exploitation of externally controlled parameters to build up a nanostructured architecture starting from larger dimensions [11]. Conversely, a “bottom-up” approach involves the growth of (sub)nanometer components (i.e., colloids, (macro)molecules, or even atoms) to produce complex nanoarchitectures [12].

The fabrication of well-ordered nanostructured materials has developed considerably in recent years, thus becoming an immensely attractive (and multidisciplinary) field of research [13–20]. In particular, nanoscopic-ordered porous architectures in the form of thin films have received great attention in the field of membrane science and micro/nanofluidics, due to the high selectivity introduced without the loss of the mechanical properties (provided by the macroporous substrate) [21–25]. Interestingly, devices based on this technology have found commercial application in separation processes involving complex matrices, such as in the clarification of beverages (i.e., milk, beer, and juices) [26], or in the selective removal of bacteria in blood [27].

Porous polymeric coatings possess the advantages of high surface area materials with a well-defined porosity [28–29], easy processability (i.e., to form molded monoliths or thin films) [30–32], and the possibility of using different synthetic routes to facilitate the incorporation of multiple chemical functionalities into the porous framework or at the pore surface [33]. The self-assembly of block copolymers is an exceptional strategy for inducing well-ordered and regular porosity in polymers [6,32]. Block copolymers (BCs) are macromolecules consisting of two (or more) immiscible homopolymer chains covalently linked together. Mesoscale nanostructures can be obtained due to the thermodynamic incompatibility of the blocks, which induce microphase separation via self-assembly, in order to minimize the contact energy between the incompatible segments forming the BCs [34]. BCs can have two different roles in the preparation of nanostructured porous materials: either as templating agents [4,6,35] or as origin of the porous framework (exploiting their self-assembly capability) [36–37]. In particular, by varying the block copolymer parameters (mostly, molecular weight, and the different blocks volume fraction) and the formulation (i.e., solvent(s) volume), it is possible to modulate the surface layer organization at the level of a few tens of nanometers.

The following paragraphs describe how well-ordered (in)organic porous coatings and membranes are obtained due to the action of BCs either as templating agents or as the source of the porogen by self-assembly. In order to guide researchers in the field of highly organized porous coatings, a detailed discussion of both approaches is presented here. In this context, different BCs are available on the market (or are eventually synthesizable), opening an infinite number of possibilities. Some properties belong to BCs (considered as a general category), whereas others are strictly correlated to the blocks forming the polymeric chains (e.g., residual functionalities, reactivity). Since the scientific literature describing the properties of BCs is extremely vast, this review will only consider (and analyze in detail) the works and the technical discussion relevant for this review. Moreover, for simplicity, only the scientific literature describing polystyrene-*block*-polyethylene oxide (PS-*b*-PEO) systems is here considered, since the knowledge gained from the PS-*b*-PEO systems can be more generally applied (and mostly valid for the other subfamilies of BCs). Additionally, among the different BCs, PS-*b*-PEO systems are very attractive due to the presence of some particular functionalities forming the two blocks (namely, the hydroxy end groups from the PEO moieties), which make this class of BCs exploitable for further functionalization reactions [38].

Therefore, with the aim of highlighting the peculiar properties of BCs, PS-*b*-PEO systems are critically discussed in this review, with a particular emphasis on their capability of growing well-ordered nanostructured porous architectures and coatings, exploitable for designing smart membranes and other devices for next future advanced applications.

### Block copolymer self-assembly: theory and application

**Figure 1:**
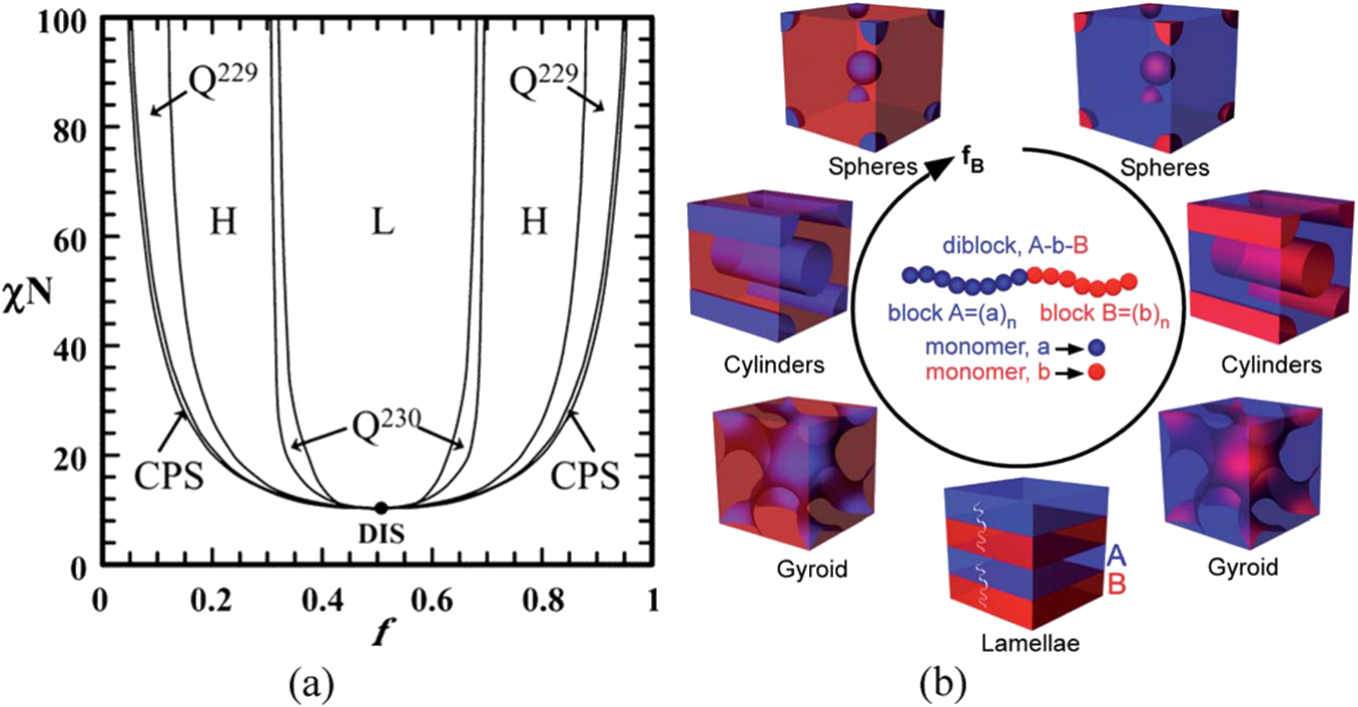
a) Phase diagram of diblock copolymer predicted by SCMF theory. Reprinted with permission from [41], copyright 2006 American Chemical Society. b) Various microdomain organization patterns of a linear AB diblock copolymers. Reprinted with permission from [42], copyright 2014 The Royal Society of Chemistry. *f*: volume fraction of one block; χ: Flory–Huggins interaction parameter; N: degree of polymerization; L: lamellae; H: hexagonally packed cylinders; Q^230^: double-gyroid phase; Q^229^: body centered spheres; CPS: closed-packed spheres; and DIS: disordered.

The self-assembly of BCs represents an exceptional strategy for inducing well-ordered and regular porosity in polymeric structures. As already mentioned in the Introduction, BCs are macromolecules made of two (or more) blocks (i.e., series of monomeric units) of homopolymer chains, thermodynamically incompatible, linked together by covalent bonds. According to the self-consistent mean field (or SCMF) theory [39], it is possible to predict the nanoscopic domain structure (i.e., spherical, cylindrical, double gyroid, and lamellar) for an AB diblock copolymer (as reported in Figure 1A) [40–42]. As indicated in Figure 1B, by increasing the volume fraction, *f,* of one of the blocks, the microdomain arrangement changed from closely packed spheres (CPSs), to body centered cubic spheres (Q^229^), to hexagonally packed cylinders (H), to bicontinuous gyroid (Q^230^, which becomes unstable at high values of segregation power χN) and lamellae (L).

The principal driving factors governing the self-assembly of BC melts is the immiscibility of the two blocks (quantified by the Flory–Huggins interaction parameter χ) that drives the system to segregate as the temperature decreases (inversely proportional), giving an order–disorder transition at a certain value of χN [43]. In detail, the Flory–Huggins model relies on the thermodynamics of polymer solutions by considering the Gibbs free energy for mixing polymer with solvents. According to the Flory–Huggins theory, to calculate the Flory–Huggins interaction parameter, it must be considered that, in a binary system, both polymer and solvent are randomly distributed in the volume, and the heat of mixing is proportional to the volume fraction of polymer segments in the volume. Hence, the free energy minimization during microphase separation favors the formation of various thermodynamically stable structures on the nanometer length scale [44]. However, microphase separation can also be influenced by the presence of a more complex formulation due to the addition of a lower molecular weight component (such as additives and homopolymers) as well as a block-selective solvent [43,45].

Table 1 reports a survey of the principal BCs used for obtaining ordered porous architectures [45–79]. In general, all microdomain arrangements predicted by the SCMF theory, except CPSs, have been experimentally verified for many different BCs. The CPS phase, which is located between the disordered state (DIS) and Q^229^ phase, has not been observed experimentally for neat BCs formulations, but it has been verified for several BCs/solvent mixtures [49,80] and/or BCs/homopolymer formulations [81–82]. The microdomain arrangement is affected by heating, since BCs can exhibit phase mixing upon heating, due to the increase in the translational (or combinatorial) entropy and subsequent decrease of the phases interaction area. It can also exhibit phase separation as a result of the thermal expansion coefficients and/or directional enthalpy (or entropy) changes, as shown in [83]. Furthermore, crystallinity is also an important parameter that can influence the domain orientation. As reported by Register and co-workers [84], there are three different levels of orientation: i) the orientation of the polymer chains within the lamella crystals, ii) the orientation of the lamella crystals within the domain structure of the block copolymer, and iii) the domain structure itself. Additionally, it is also possible to rationalize the crystallization behavior in BCs considering the degree of miscibility of the components. This suggests that the microphase separation morphology is also affected by the composition of the blocks forming the BCs. Semicrystalline BCs give two different crystallization modes: break-out crystallization and confined crystallization (the last one typical when the crystalline block is the minor component); for a detailed discussion, please refer to [85]. In this context, BCs containing crystalline blocks (such as PE and PEO domains) show two different chain-folding orientations that give different domain-structure orientation: perpendicular folding, whose domains are perpendicularly oriented with respect to the lamellar layer, and parallel folding, with domains parallel with respect to the lamellar layer [86]. Perpendicular folding occurs when a low molecular weight BC crystallizes from a disordered phase (or from a poorly segregated phase). With increasing molecular weight, the interfacial area per block junction increases, inducing parallel folding (the most thermodynamically stable form) [87]. The removal of the sacrificial component (SC) to obtain the final polymeric porous material can be performed using various etching procedures, such as plasma oxidization [53], electron beam curing, as well as laser and/or selective decomposition (as reported in Table 1).

**Table 1:** Block copolymers (BCs), sacrificial components (SC), and the microstructures of the porous polymeric architectures.

BCs^a^	SC	etching conditions	microstructure	ref.

1,2-PB-*b*-PDMS	PDMS	tetrabutylammonium fluoride in THF	H	[46]
P2VP-*b*-PI, PI h.	PI	ozonolysis	Q^230^	[47–48]
P3DDT-*b*-PLA	PLA	NaOH	H	[49]
(P3HT-NH_3_^+^)-*b*-(PS-SO_3_^−^)	PS-SO_3_^−^	acetate, triethylamine	n.d.	[50]
PFS-*b*-PLA	PLA	NaOH	H, Q^230^	[51–52]
PI-*b*-PS, PI h., PS h.	PS h.	hexane	Q^230^	[53]
PFS-*b*-PMMA	PMMA	UV radiation	Q^230^	[54]
PE-*b*-PEP, PE h., PEP h.	PEP h.	THF	Q^230^	[55]
PE-*b*-PS	PS	fuming HNO_3_	H, Q^230^	[56–57]
PLA-*b*-P(N-S)	PLA	NaOH	H	[58]
**PS-*b*-PEO**	PEO	HI or heating	H, Q^230^	[59–63]
**PS-*b*-PEO, resorcinol**	resorcinol	2-propanol	H, Q^230^	[45,64]
PS-*b*-PLA	PLA	NaOH or HI	H, Q^230^	[65–69]
PS-*b*-PDMS	PS	O_2_ plasma treatment	H	[70]
PS-*b*-PMMA	PMMA	UV radiation	H, Q^230^	[71–74]
PS-*b*-PMMA, PMMA h.	PMMA h.	CH_3_COOH	H	[75–76]
PSTPA-*b*-PLA	PLA	NaOH	H	[77]
PS(BCB)-*b*-PMMA	PMMA	UV radiation	H	[78]
PS(BCB)-*b*-PLA	PLA	NaOH	H	[79]

^a^h.: homopolymer; 1,2-PB: 1,2-poly(butadiene); P2VP: poly(2-vinylpyridine); P3DDT: poly(3-dodecylthiophene); P3HT-NH_3_^+^: aniline chain-end-functionalized poly(3-hexylthiophene); PDMS: poly(dimethylsiloxane); PE, poly(ethylene); PEO: poly(ethylene oxide); PEP: poly(ethylene-*alt*-propylene); PFS: poly(ferrocenylsilane); PI: poly(isoprene); PLA: poly(lactic acid); PMMA: poly(methyl methacrylate), P(N-S): poly(norbornenylethylstyrene-*s*-styrene); PS: poly(styrene); PS(BCB): poly(styrene-*r*-benzocyclobutene); PS-SO_3_^−^: sulfonic acid chain-end-functionalized poly(styrene); PSTPA: poly(styrene) containing triphenylamine side group.

Hozumi and co-workers [74] investigated the removal of poly(methyl methacrylate) (PMMA) domains in a PS-*b*-PMMA copolymer film by using 172 nm vacuum-ultraviolet (VUV) light. In this case, the selective etching of activated oxygen molecules generated by the VUV radiation towards the two blocks (PS and PMMA) allowed for the preferential decomposition of PMMA and the consequent formation of a PS nanoporous network. The modulation of the irradiation time and pressure caused chemical and physical modifications of the PS nanostructures, since the complete removal of PMMA phase produces a hydrophobic PS surface whereas an irradiation at a pressure of 103 Pa caused the partial decomposition of the PS matrix with the modification of the material pore size and structure.

By focusing on PS-*b*-PEO copolymers, Mao et al. [59] demonstrated that the chemical etching of the minority component leads to the formation of a well-ordered nanoporous system by selective removal of the PEO domains by simple ether cleavage by washing with aqueous hydrogen iodine. This strong acid was selected for its specific debonding reactivity toward the aliphatic ether functionalities forming the PEO chains [88]. Furthermore, they tried also to obtain a monolithic nanoporous material with nanochannels of ≈10 nm width [61]. Unfortunately, the extremely harsh conditions due to the aqueous hydrogen iodine make this solution difficult to apply in thin films or coatings. In the work of Zhang and co-workers [60], a specific PS-*b*-PEO copolymer containing a cleavable juncture (namely, triphenylmethyl (trityl) ether) between the two blocks PS and PEO was prepared. This solution guarantees that acids under mild conditions can easily cleave the linkage between the blocks without affecting the block’s self-organization.

Based on almost the same principle, nanoporous thin films with well-ordered cylindrical pores were obtained by preparing metallo-supramolecular block copolymers (where the two different polymeric blocks are linked via metal–ligand complexes) [63]. In this particular case, the approach consists of firstly, the self-assembly of the metallo-supramolecular block copolymer, forming a well-ordered thin film, and secondly, the opening of the metallo-complex via redox reaction, extracting the PEO moieties. In this study, the metallo-complex selected is Ru(II)-terpyridine bis-complex. By washing the film with a Ce(IV)-containing acid solution, the Ru(II) complex between the blocks is oxidized into Ru(III) that is able to form only a monocomplex with the terpyridine ligands, thus breaking one metallo-organic bond. The aqueous environment favored the extraction of the freely accessible, soluble PEO and Ru-PEO moieties, leaving both PS and Ru-PS moieties to form the nanoporous polymeric matrix.

It has been demonstrated that by controlling the annealing procedure and the humidity, it is possible to control the orientation of the PEO cylindrical domains within the PS thin film [89–90]. In particular, at high humidity conditions, it has been found that PEO cylindrical domains are vertically (perpendicular) oriented with respect to the thin film surface, whereas at low humidity conditions, the PEO domains are horizontally (parallel) oriented [90].

**Figure 2:**
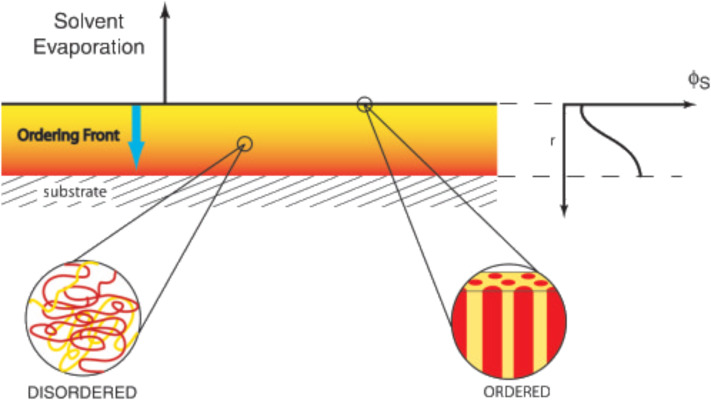
Schematic representation of the solvent evaporation in a thin film made by block copolymer. At the surface, the concentration of the solvent is low and the copolymer undergoes a well-ordered microphase separation. A gradient in the concentration of the solvent (as a function of the film thickness, *r*) is established perpendicular to the surface. In the bulk, the concentration of the solvent is high, thus block copolymer chains forming the interior part of the film are disordered. As the solvent evaporates, an ordered front propagates through the film thickness, producing a high vertically ordered cylindrical microdomain orientation. Reprinted with permission from [90], copyright 2004 Wiley-VCH.

As reported previously [90], the order achieved in thin films made by PS-*b*-PEO copolymers depends only on either the solvent casting or the solvent vapor annealing conditions, and not the substrate. Furthermore, the presence of the solvent in these polymeric systems enhances the disorder degree within the polymeric chains since it mediates also nonfavorable interactions within the polymeric chains, working as plasticizers (affecting also the glass transition temperature value). When the evaporation phenomenon takes place at the film surface, microphase separation occurs and long-range lateral order is reached (as depicted in Figure 2). This way, a difference in terms of orientation is generated between the surface (i.e., low content of solvent, ordered system) and the bulk (i.e., high content of solvent, disordered system) of the polymeric film. However, as the solvent evaporates, the ordering front propagates through the films, thus extending the ordered microdomain growth following the solvent gradient direction (namely, perpendicular to the surface).

**Figure 3:**
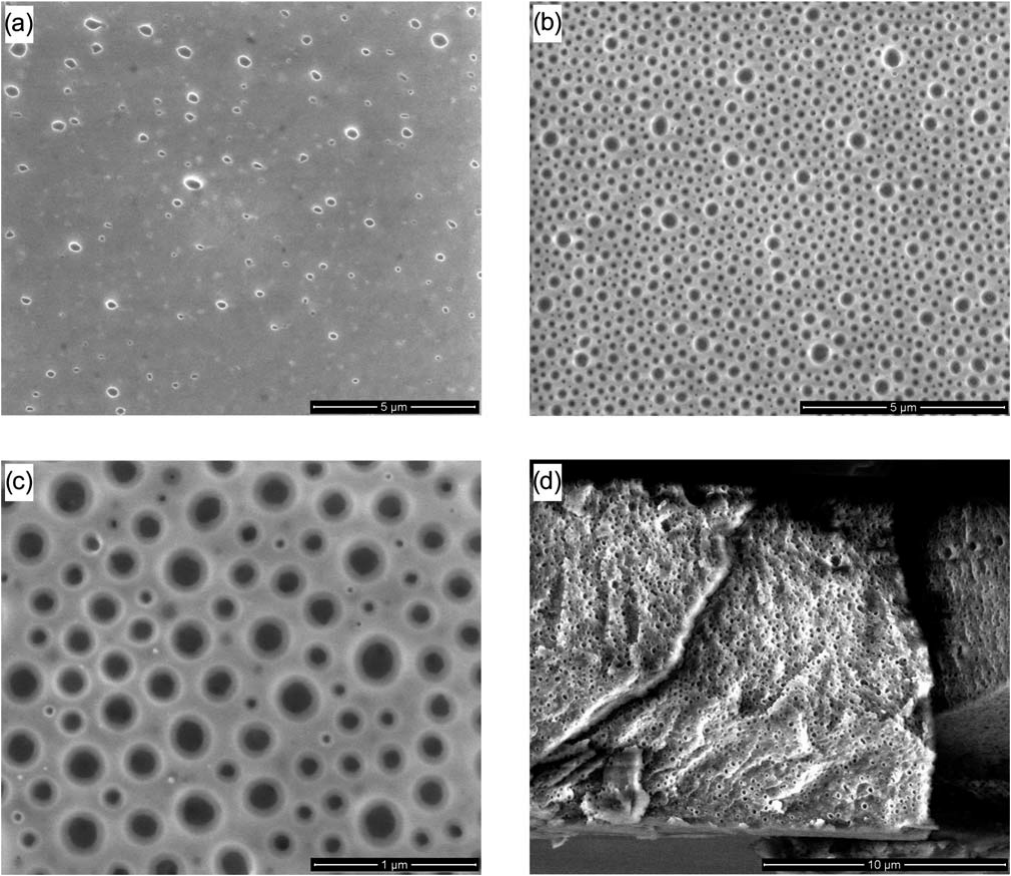
SEM micrographs of PS-*b*-PEO films after heating at 90 °C and washing with water. a) PS-*b*-PEO (18.5-*b*-5.0 kg mol^−1^). b) PS-*b*-PEO (10.6-*b*-5.0 kg mol^−1^) at low magnification. c) PS-*b*-PEO (10.6-*b*-5.0 kg mol^−1^) at high magnification. d) Freeze fracture cross section PS-*b*-PEO (10.6-*b*-5.0 kg mol^−1)^. Reprinted with permission from [62], copyright 2011 The Royal Society of Chemistry.

Since PEO is water soluble, it can be easily removed by simply heating and washing with water. In the work of Glassner et al. [62], they reported the synthesis of PS-*b*-PEO copolymers by coupling the reversible addition fragmentation chain transfer (RAFT) polymerization and the hetero Diels–Alder cycloaddition followed by subsequent retro-hetero Diels–Alder mechanisms by a heating/washing procedure. In this study, diblock copolymers are drop cast onto silicon wafers as substrates from a diluted chloroform solution. The SEM images in Figure 3 report the morphology of PS-*b*-PEO films after heating at 90 °C and washing with water. The formation of pores due to the removal of PEO domains is clearly demonstrated. Additionally, by increasing the amount of PEO moieties within the block copolymers (and/or consequently reducing the PS ones) a marked intensification in porosity is observed within the entire thickness of the film (thus suggesting that this phenomenon is not surface-limited).

In general, there are different methods for controlling the final morphology in the self-assembled BC coating. Some of these methods require the use of solvents, such as in the work of Karunakaran et al. [91] where the possibility of producing isoporous PS-*b*-PEO-based membranes by separating layers using water at room temperature as coagulant was reported. In this study, the PS-*b*-PEO BC membranes were obtained by a phase-inversion process starting from a solution of a DMAc/THF/sulfolane solvent mixture and by immersing the casting films in deionized water at room temperature. By comparison with analogous PS-*b*-P4VP membranes, the results obtained for PS-*b*-PEO membranes evidenced that the pore dimensions of the PS-*b*-PEO membranes are not affected by the pH change (in contrary to PS-*b*-P4VP). Additionally, since the membranes with PEO moieties present hydroxy end groups, the pore size can be tailored by further functionalization of the hydroxy functionality, thus making PS-*b*-PEO membranes attractive for several applications.

Other methods require the introduction of swelling agents (i.e., agents for increasing the microdomain dimensions) [92] as well as additives able to affect the microdomain orientation or act as a sacrificial component [64,93]. The inclusion of a homopolymer in the formulation is also a possible route to introduce particular effects on domain orientation and stability. As reported by Zhu et al. [93], PS-*b*-PEO BCs can be blended with PS homopolymers of different molecular weights to obtain a high molecular weight PS homopolymer with “hard confinement”, whereas the low molecular weight one led to “soft confinement”. Thus the thermodynamic stability of the PEO domains can be modulated in a controlled fashion.

**Figure 4:**
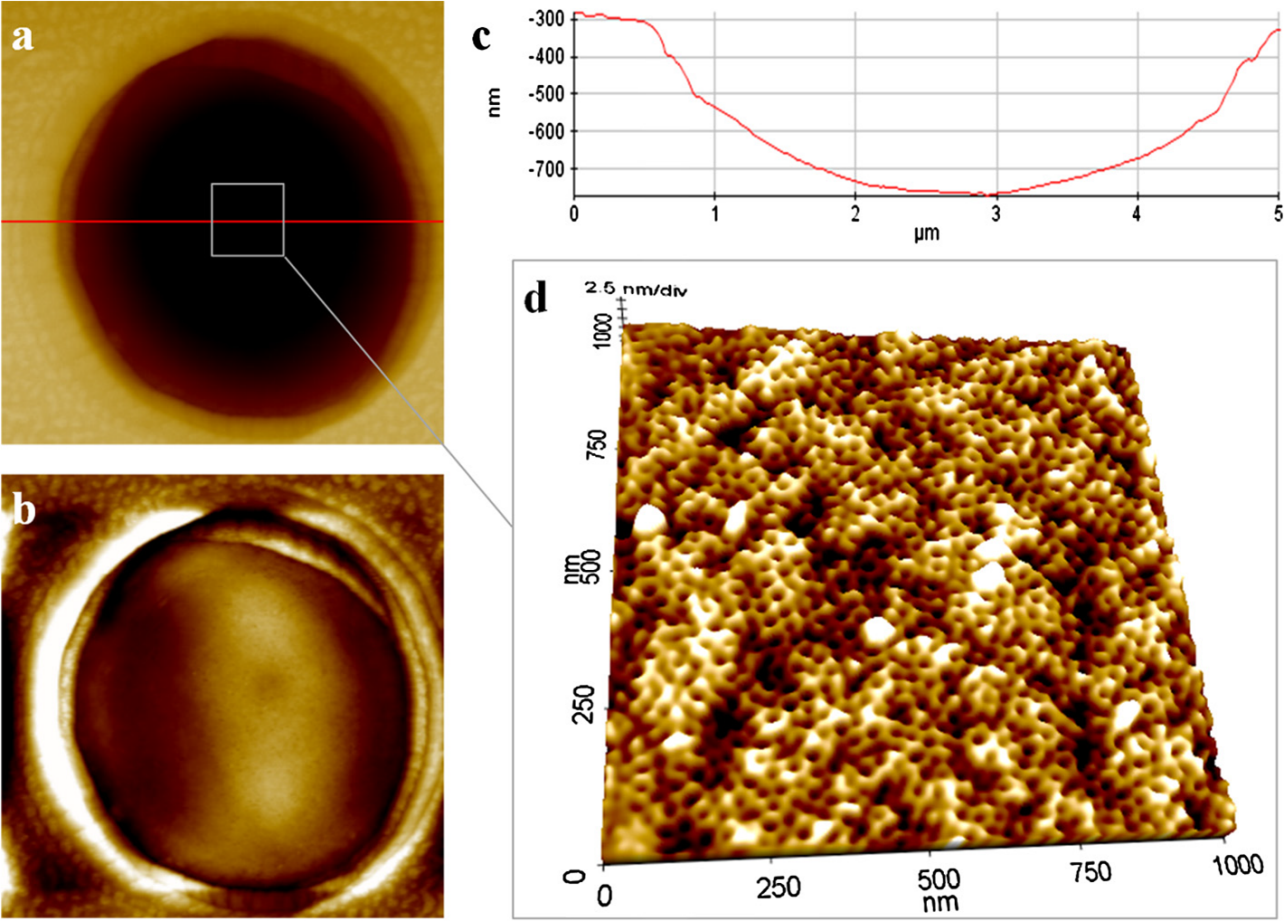
a) Atomic force microscopy (AFM) images of a composite nanoporous membrane: detail of the Si microsieve with the nanoporous polymeric membrane on top. b) Corrected AFM image showing the coverage of the Si macropore by the polymeric membrane. c) AFM profile corresponding to the horizontal line in a). d) 3D AFM image showing the morphology of the nanoporous layer within the macropore. Reprinted with permission from [64], copyright 2014 Elsevier.

In a previous study [64], solutions containing PS-*b*-PEO block copolymers were spin-coated onto a macroporous substrate (namely, silicon microsieves with pores of 5 µm width). Since the goal was to obtain a perpendicular cylindrical morphology, a possible technical solution is the addition of small molecules (or salts) able to stabilize a preferentially interaction with one of the blocks, through the formation of hydrogen bonding between the small organic molecules and one of the copolymer blocks. This way, it is possible to favor the normal orientation of the cylindrical nanodomains [94]. In this paper, resorcinol is selected as the orienting molecule to direct the orientation of the ethylene oxide cylindrical domains in PS-*b*-PEO copolymers. UV light irradiation was used to crosslink the PS matrix and photodegrade the PEO domains. Afterwards, several washing techniques were tested to selectively remove the resorcinol together with the PEO moieties (selective cleavage), where 2-propanol was determined to be the best solvent. As reported in Figure 4, the nanoporous thin membrane (i.e., pore size ≈20 nm) adheres to the macroporous substrate without any discontinuities. As reported in Figure 4d, the desired vertical alignment of the nanoporous system is maintained, even inside the substrate macropore. Additionally, transport studies were also performed, selecting two different target molecules. Size-selective sensitivity was confirmed, thus suggesting the possible application of these coatings in membrane technology for increasing the controlled transition of chemicals in separation processes.

### Block copolymers and templating: theory and application

**Figure 5:**
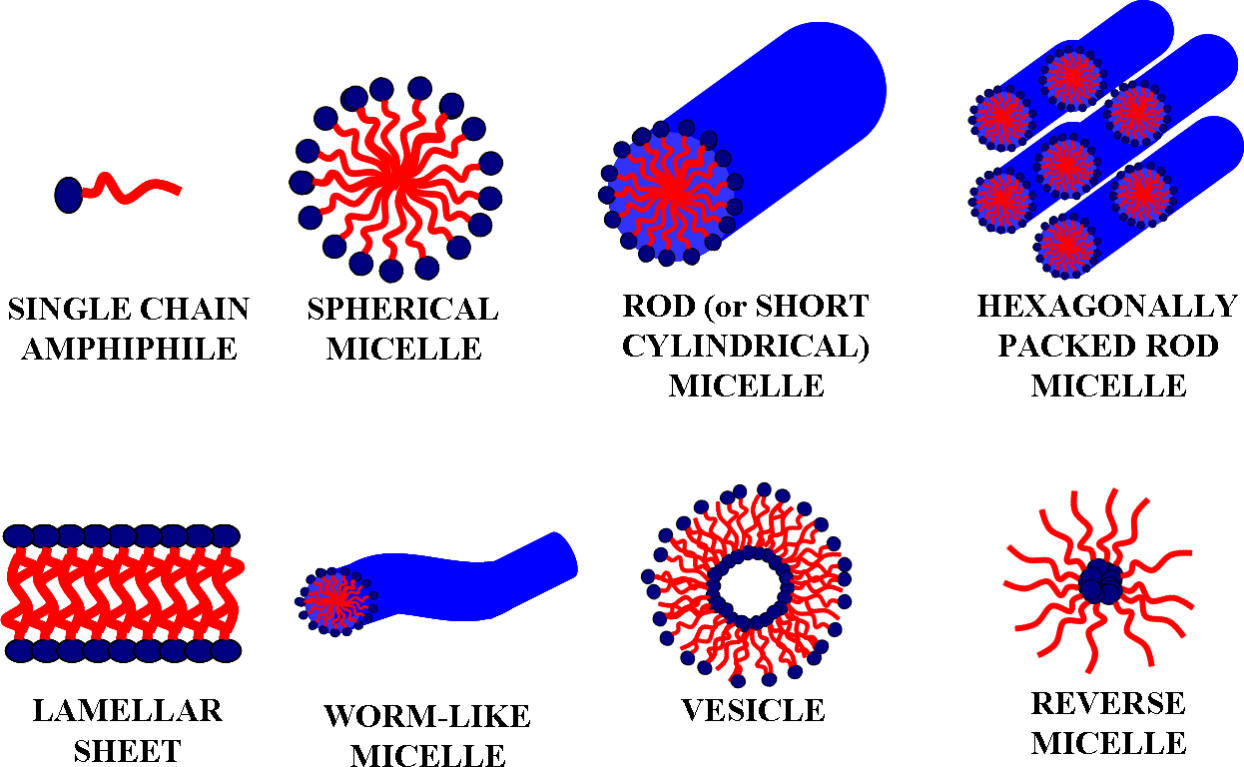
Schematic representation of different micellar architectures. Hydrophilic polar heads are indicated in blue, and hydrophobic non-polar tails are drawn in red. Reprinted from [22] (“Selective porous gates made from colloidal silica nanoparticles”); source published under Creative Commons Attribution 2.0 license, https://creativecommons.org/licenses/by/2.0/; copyright the authors.

Amphiphilic BCs (such as PS-*b*-PEO) are a subcategory of copolymers which can self-organize to form supramolecular aggregates with specific shapes, such as: spherical, rod (or short cylindrical), hexagonally packed rod micelles, reverse micelles as well as worm-like structures, lamellar sheets, and vesicles (Figure 5). As mentioned previously, the thermodynamic incompatibility between the blocks forming the polymer chains is the driving force behind the formation of such nanostructures [4,35]. In this context, this peculiar characteristic can be coupled with sol–gel processes to produce well-ordered oxidic architectures [95–96].

The sol–gel process involves various chemical reactions such as hydrolysis, condensation, and consequently, polymerization involving the monomers (for inorganic systems, either metal alkoxides or metal chlorides) that evolves forming a colloidal solution (sol) and subsequently a stable network (gel) of polymerized particles. The byproducts of these polycondensation reactions are water and alcohol, depending on the precursor selected. Even if the principles behind the sol–gel reaction are very simple, several parameters can influence the resulting architecture of the designed material, such as the type of catalyst (i.e., acid or base), temperature conditions and atmosphere, reaction medium (i.e., either water or other non-aqueous solvents), and so on. For a detailed discussion concerning the principles behind sol–gel chemistry, please refer to [4].

In order to enhance the structural control in oxidic systems (and in particular by modulating the porosity organization), one possibility is to exploit the templating action of amphiphilic BC supramolecular structures working as structural directing agents (SDAs) [97–98]. This way, the final material corresponds to the negative replica of the SDA. There are two principal templating methods: hard templating (or nanocasting) and soft templating [97]. Hard templating involves an exotemplating approach, where the precursor solidifies within the solid SDA. In soft-templating, an endotemplating approach is used where the precursor starts to solidify around the porogens (which remain in the liquid state).

Regarding the hard-templating route, the preparation of titanium structures on block copolymer films has been recently reported, where the titanium assembly is driven by the microphase separation of the PS-*b*-PEO layer underneath [99]. When the titanium coating procedure is performed by electron beam evaporation onto a previously self-assembled PS-*b*-PEO substrate, Ti preferentially deposits and diffuses inside the PS matrix, thus leaving the PEO domains visible (and forming a porous structure). Analogous results were also obtained for cobalt onto PS-*b*-PEO [100].

Concerning the soft-templating route, usually the most diffuse porogens used are surfactants: small molecules characterized by having both polar (head) and apolar (tail) parts linked together by chemical bonds [101]. Analogous to surfactants, even amphiphilic block copolymers (such as the PS-*b*-PEO ones) can be used as templating agents [4].

**Figure 6:**
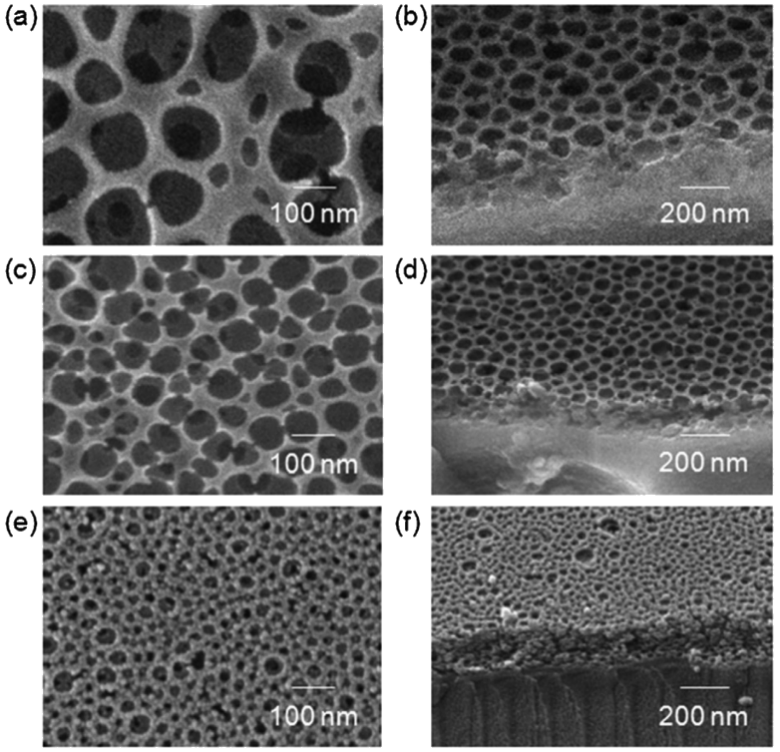
Top-view (left) and tilted 60° (right) SEM micrographs of PS_962_-*b*-PEO_3409_ (a, b), PS_563_-*b*-PEO_1614_ (c, d) and PS_385_-*b*-PEO_1205_ (e, f) soft-templated titania films. Reproduced with permission from [105], copyright 2011 The Royal Society of Chemistry.

As reported by Yu and co-workers [102], PS-*b*-PEO copolymers were used for the production of mesoporous silica films where a cubic close-packed spherical system was obtained by solvent evaporation induced self-assembly (EISA) process. Different pore sizes can be obtained by changing the block length in the soft templates. By coupling the spin-coating deposition technique with the soft-templating approach, mesoporous silica coatings were obtained using PS-*b*-PEO block copolymers as SDAs [103]. In this study, by modulating the starting composition (in particular, the hydrophilic/hydrophobic solvent ratio), a transition from stacked spherical pores to worm-like structures to spherical dense particles was reached due to the minimization of the surface free energy [104]. Analogously as for silica, even titania can be produced with closed spherical pores within the oxidic structures by using high molecular weight PS-*b*-PEO copolymers (as shown in Figure 6) [105].

Organic–inorganic PS-*b*-PEO/TiO_2_ hybrid nanostructured coatings can also be produced by spin-coating deposition followed by calcination in order to obtain a nanostructured titania layer [106]. The thermal degradation of the organic polymeric template was successfully achieved without causing a collapse of the titania nanoarchitecture. The driving force behind these systems is the polar affinity between titania and the PEO domains (this is another advantage of this class of BCs, namely PS-*b*-PEO). By changing the titania precursor (i.e., TTIP) and the BC volume ratio, it was possible to drive the self-organization of the PEO domains, and consequently, the titania nanostructure. AFM images reported in this study show that after thermal treatment, a mesoporous titania coating is obtained where the spherical pore systems correspond to the PS spherical domains in the hybrid film before calcination. Depending on the formulation parameters and following the same procedure, even titania worm-like structures were obtained.

**Figure 7:**
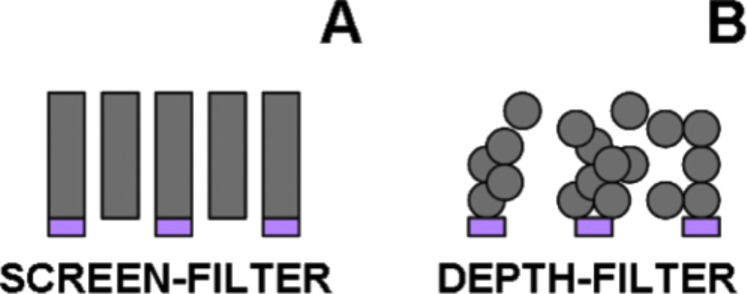
Schematic cross section of a screen filter (A) and a depth filter (B). Reprinted with permission from [4], copyright 2017 Elsevier.

In this context, the exploitation of such functional porous coatings is very appealing from the membrane technology viewpoint by direct deposition onto a macroporous substrate (whose role is to guarantee the necessary mechanical resistance), thus forming functional filtering systems [107]. Among the different porous systems, a distinction can be realized between screen filters (well-ordered vertically aligned pore sieves, see Figure 7A) and depth filters (disordered tortuous pore systems, see Figure 7B). The main difference between these two systems is the principle behind the sieving method: screen-filter separation is based on size exclusion, whereas depth-filter separation is based also on the interactions between the material forming the functional membrane and the target molecule being separated/isolated.

In our recent study [22], colloidal silica nanoparticles (produced by using PS-*b*-PEO block copolymers as templates) are deposited via spin-coating onto a macroporous silicon-based substrate, forming a depth-filtering system (i.e., interparticle voids of 15–200 nm). In order to evaluate the selectivity of this porous membrane, two cationic (macro)molecules were selected as target probes: methylene blue (a dye of 0.5 nm in width) and the protein RNAse (3.8 nm), respectively. The results evidenced that the diffusion of the protein is more restricted as compared to the dye, suggesting a steric selectivity of the depth-filtering system analyzed. In addition, by applying an external electrical stimulus, the migration of both probes was registered with an increasing transport rate of the two chemical species.

### Critical considerations

Membrane-mediated processes are widely considered one of the most promising solutions to be exploited for industrial separation and microfluidic dosing operations [108–110]. Currently, the integration of BCs (with their particular self-organizing capacity) is playing an increasing role in the development of nanoscale-controlled porous systems [4,111]. In this review, several case studies have been presented, highlighting the potential exploitation of PS-*b*-PEO amphiphilic block copolymers for designing both oxidic and polymeric nanoporous membranes. For the sake of comparison, the choice of the best technical solution strongly depends on the matrix properties forming the advanced functional coatings.

BC-based polymeric nanoporous coatings guarantee both high morphological flexibility and a very narrow pore size distribution [112]. Additionally, such polymeric membranes are good candidates for the selective separation of (bio)molecules and microorganisms [75] as well as in water purification treatment due to their high selectivity, permeability, fouling resistance and mechanical strength. Eventually, further functionalizing can enhance the BC selectivity/affinity toward a particular target probe. Unfortunately, these advantages are partially annulled by a few drawbacks related to the production of these starting BCs (i.e., high cost, economic concern) and the organic solvents (which are sometimes not ecologically friendly) necessary for guiding the self-assembly (i.e., environmental concern).

On the other hand, the BC-templated porous inorganic coatings present several advantages and disadvantages depending on the templating technique selected. Soft templating, in particular, is the more versatile technique as it allows very complex morphologies to be obtained, which are almost impossible to obtain using nontemplated sol–gel processes [113]. Besides this fundamental advantage, for both templating methods, the principal critical step is the removal of the porogen without losing the designed nanostructure organization [4]. In general, this procedure is a very complex route that requires either strong acids/bases or selective organic solvent washing (not easy to handle or environmentally friendly), or thermal treatments (which risks the formation of carbonaceous residues entrapped within the inorganic porous architecture) [114]. Concerning hard templating, the structural compatibility between the SDA and the material precursor should be considered in a way that avoids undesirable voids or cracks induced by the solid templates [115].

Furthermore, the continuous development of novel functional substrates with highly controlled porosity is an increasing field of research that involves worldwide experts from both academia and industry. According to the works summarized in this review, the highly ordered nanostructures obtainable by exploiting the peculiar properties of BCs have found ample use in membrane technology, even if there are still economic and environmental concerns that need to be overcome. Among the different types of BCs, this review focused on the main results obtained for PS-*b*-PEO in particular. Due to the functionalities forming the two blocks, PS-*b*-PEO is a very attractive material for the production of (in)organic porous coatings and thin films to be exploited in membrane science and in dosing of chemicals. Additionally, the hydroxy end-functional groups forming the PEO domains constitute an interesting intermediate step for further functionalization, thus opening up even more application possibilities.

## Conclusion

In this manuscript, the fabrication of well-ordered nanostructured porous coatings by means of block copolymers was reviewed. The most recent advances were summarized in order to provide a simple toolbox to follow for the preparation of (in)organic isoporous thin coatings exploitable for the development of novel well-ordered devices for membrane science and microfluidics applications. The interesting properties of amphiphilic block copolymers (taking PS-*b*-PEO as a model BC) in terms of self-assembly and templating action were highlighted, encouraging their use in important fields of research, such as in sensing, dosing, and separation processes. In this context, it is important to point out that membranes already find commercial application in many separation processes of complex matrices, such as in the clarification of beverages (i.e., milk, beer, and juices), the remediation of polluted water, or in the selective removal of bacteria and viruses from bloods. The main advantage of this technique is that membrane technology is a simple, robust, and well-consolidated technique that guarantees high performance and easy scale up, as compared to other more fascinating (albeit still at the laboratory scale) approaches, such as the use of magnetic adsorbing materials [116] or advanced oxidized processes [117–118]. On the other hand, future research should be focused on overcoming the economic and environmental concerns related to the exploitation of these block copolymers for designing nanostructured materials/coatings. To the best of our knowledge, there is no single, feasible technical solution available. The integration of consolidated processes with novel and more sustainable solutions could be a path forward, but the discussion is still open.
